# Biologging of body temperature in a desert ungulate reveals water use, sex-specific seasonal thermoregulation and heterothermy-associated mortality

**DOI:** 10.1093/conphys/coag056

**Published:** 2026-08-06

**Authors:** Paige R Prentice, Hendrik J Combrink, Christina M Aiello, Holly K Arnold, Brianna R Beechler, Anna E Jolles, Clinton W Epps

**Affiliations:** Department of Fisheries, Wildlife, and Conservation Sciences, Oregon State University, 104 Nash Hall, Corvallis, OR 97331, USA; California Department of Fish and Wildlife, Wildlife Branch, 1010 Riverside Parkway, West Sacramento, CA 95605, USA; School of Natural Resources and the Environment, The University of Arizona, 1064 East Lowell Street, Tucson, AZ 85179, USA; Department of Fisheries, Wildlife, and Conservation Sciences, Oregon State University, 104 Nash Hall, Corvallis, OR 97331, USA; Wildlands Network, P.O. Box 243, Salt Lake City, UT 84110, USA; Department of Biomedical Sciences, Carlson College of Veterinary Medicine, Oregon State University, Magruder Hall 700 SW 30th St, Corvallis, OR 97331, USA; Department of Biomedical Sciences, Carlson College of Veterinary Medicine, Oregon State University, Magruder Hall 700 SW 30th St, Corvallis, OR 97331, USA; Department of Biomedical Sciences, Carlson College of Veterinary Medicine, Oregon State University, Magruder Hall 700 SW 30th St, Corvallis, OR 97331, USA; Department of Integrative Biology, Oregon State University, 2403 Cordley Hall, Corvallis, OR 97331, USA; Department of Fisheries, Wildlife, and Conservation Sciences, Oregon State University, 104 Nash Hall, Corvallis, OR 97331, USA

**Keywords:** Biologging, body temperature, desert bighorn sheep (*Ovis canadensis nelsoni*), heterothermy, survival, thermoregulation, water consumption

## Abstract

The inability to properly thermoregulate can be an early sign of physiological stress and indicate poor fitness in mammals. Advances in biologging technology enable continuous monitoring of core body temperature in free-ranging animals, offering insights into thermoregulation. Arid environments are characterized by extreme daily and seasonal fluctuations in temperature and limited water availability. As such, desert animals balance energetic demands of self-maintenance and reproduction against thermoregulatory constraints. In this study, we deployed reticulum implant transmitters (biologgers) in 43 desert bighorn sheep (*Ovis canadensis nelsoni*) across six Mojave Desert mountain ranges from November 2020 to December 2021, coinciding with a severe drought. Our aim was to (i) detect and assess frequency of water consumption, (ii) characterize seasonal, population-level and sex-specific patterns in daily body temperature metrics (minimum, maximum, median and amplitude) and (iii) test whether body temperature variability predicted mortality risk. Desert bighorn typically only rely on surface water during the hotter months, but biologgers provided high-resolution, year-round evidence of water consumption, providing novel insights into water usage during a drought year. Females exhibited greater thermal flexibility than males through increased body temperature variation via lower daily minima in the spring and higher daily maxima in the summer. Males were able to lower their body temperature during the summer mating season without increasing body temperature variation. Individuals that died exhibited significantly greater body temperature variability via lower daily minima preceding death than did survivors during corresponding periods—an early indicator of increased mortality risk in desert bighorn. Continuous core temperature monitoring via biologgers thus offers a powerful tool for linking physiological state to fitness in the field of conservation physiology.

## Introduction

Extreme heat events and water scarcity have negatively affected ecosystems, wildlife and livestock across the planet over the last 50 years, and these trends are predicted to intensify (IPCC, 2023). Consequently, many animals face thermal stress and potentially reductions in fitness (Wilson and Krausman, 2008; Ruthrof et al., 2018; Calleja-Agius et al., 2021). The ability to regulate body temperature is an essential component of homeostasis in vertebrates. This complex process, known as thermoregulation, involves both physiological and behavioural mechanisms that allow endotherms to maintain core body temperatures within an optimal range despite environmental fluctuations (Scholander *et al.*, 1950b; Schmidt-Nielsen, 1964). Heat is primarily generated through metabolism and dissipated via dry heat exchange (convection, radiation and conduction) and evaporative cooling, with morphology further influencing insulation and heat loss (Cain et al., 2006; Mitchell et al., 2018). These factors collectively define the optimal range of ambient temperatures where metabolic energy expenditure remains minimal (Porter and Kearney, 2009). When temperatures exceed the upper limits, mammals ultimately rely on evaporative cooling and increased water intake, and when temperatures are below the optimum range mammals require increased metabolic heat production (Mitchell *et al.*, 2018). Metabolic heat production is further modulated by the circadian system, which in turn generates a daily body temperature rhythm (Refinetti, 2020). Early researchers believed endotherms had relatively static thermoregulatory windows (Scholander *et al.*, 1950a); however, research has since demonstrated that the endothermic capacity for adaptive thermoregulation varies widely among species, depending on their ecological niche and evolutionary history (Angilletta *et al.*, 2010; Boyles *et al.*, 2011b).

Adaptive thermoregulation to maintain optimal body temperature requires energy trade-offs with other biological functions, such as growth and reproduction, that can result in fluctuations, or variation, in body temperature (heterothermy; Boyles *et al.*, 2011a; Hetem *et al.*, 2016). For example, increased heterothermy was associated with fewer pregnancies in wild rabbits, suggesting fitness trade-offs which may be induced by energy and water deficits (Maloney *et al.*, 2017). Other studies on desert mammals have documented that reduced water intake and dehydration are associated with increased daily maximum body temperatures (Schmidt-Nielsen *et al.*, 1956; Fuller *et al.*, 2021) and consequently increased heterothermy (Hetem *et al.*, 2010). Similarly, poor body condition resulting from disease, starvation or dehydration has been associated with increased heterothermy in large mammals, consistent with the interpretation that heterothermy reflects energetic compromise (Hetem *et al.*, 2016; Botha *et al.*, 2023). This growing body of literature highlights the important role continuous body temperature data play in allowing researchers and managers to better understand the physiological responses of free-ranging mammals *in situ*.

The domestic livestock industry has extensive data on ungulate body temperature and often relies on it as an indicator of health and physiological status, aiding in the detection of ovulation, infection and heat stress (Mader *et al.*, 2010; Boehmer *et al.*, 2015; Carabaño *et al.*, 2016; Wijffels *et al.*, 2020; Baida *et al.*, 2021). In domestic cattle, chronic heat stress can result in reduced milk production and in severe cases may cause permanent physiological alterations preventing recovery to pre-heat stress production rates (Collier *et al.*, 2019). Advancements in biologging technology via devices such as vaginally implanted transmitters, subdermal transmitters and reticulum implant transmitters (RITs) have made monitoring continuous body temperature and the impacts of physiological stressors more accessible. These large, high-resolution datasets provide critical insights into the thermoregulatory patterns of both domestic and free-ranging ungulates.

Different body temperature biologging devices have their own benefits and limitations and require an initial capture and animal handling. RITs are particularly advantageous for ruminants, as they continuously transmit data to a recoverable GPS collar—while the collar eventually detaches, the RIT remains in the reticulum for the animal’s lifetime (Signer *et al.*, 2010). Research indicates RITs accurately reflect body temperatures in domestic cattle (Beatty *et al.*, 2008b; Boehmer *et al.*, 2015), domestic sheep (Beatty *et al.*, 2008a; Baida *et al.*, 2021; Vesterdorf *et al.*, 2022), free-ranging alpine ibex (Signer *et al.*, 2010) and free-ranging moose (Herberg *et al.*, 2018). In domestic sheep, RIT temperatures consistently register 0.5 ± 0.1°C above core body temperature (Beatty *et al.*, 2008a; Vesterdorf *et al.*, 2022). In addition, because RITs are in the reticulum, they are subject to transient cooling during water consumption which may necessitate careful data processing to distinguish temperature fluctuations due to drinking versus changes in core temperature (Vesterdorf *et al.*, 2022). This ability to detect water consumption may also produce high-resolution data to better understand how frequently individuals consume water, a topic of particular interest in free-ranging desert ungulates (Cain *et al.*, 2008b; Longshore *et al.*, 2009 ; Glass *et al.*, 2022).

Desert bighorn sheep (*Ovis canadensis nelsoni*; hereafter desert bighorn) inhabit rugged, arid landscapes characterized by extreme temperature fluctuations, limited surface water and unpredictable food resources. Adapted to some of the hottest and driest places on Earth, desert bighorn exhibit behavioural thermoregulation through crepuscular activity, and/or using shaded and cooler microhabitats during peak heat (Cain et al., 2006, 2008a, 2008b). Seasonal precipitation is critical to maintain forage quality (Wehausen, 1995), and the spatial and temporal variation in precipitation results in a long lambing season (majority February–April; Rubin *et al.*, 2000; Wehausen, 2005). During the cooler months, forage alone may be enough to meet daily water requirements (Gedir *et al.*, 2016), but in the summer most individuals rely on surface water or moisture-storing succulents such as barrel cactus (Longshore *et al.*, 2009; Glass *et al.*, 2022). Surface water is unevenly distributed across the landscape in the form of natural springs, tinajas (natural rock basins) and wildlife water developments (i.e. artificial water sources; Rich *et al.*, 2019), but natural water sources are becoming more ephemeral due to climate change (Dekker and Hughson, 2014) and groundwater pumping (Parker *et al.*, 2021). Desert bighorn routinely increase their reliance on surface water during hot periods (Longshore *et al.*, 2009; Glass *et al.*, 2022), yet year-round visitation rates remain poorly quantified—information that is increasingly relevant to wildlife managers under warmer and more frequent drought conditions. Drinking frequency is of biological and management interest as wildlife water developments must be appropriately monitored and maintained to provide adequate water to desert wildlife, particularly during drought conditions and in vulnerable populations.

The desert bighorn populations in the Mojave Desert of California are small, mostly <100 individuals, and occupy isolated mountain ranges separated by expanses of open desert (Bleich *et al.*, 1990). Although animals are known to make occasional or seasonal movements among nearby mountain ranges (Epps *et al.*, 2007; Dekelaita *et al.*, 2023), connectivity is restricted by anthropogenic barriers such as interstate highways (Epps *et al.*, 2005, 2010; Aiello *et al.*, 2023, 2024), and climatic refugia are limited to only a few higher elevation ranges (Epps *et al.*, 2004, 2006). In the 20th century, numerous population extinctions occurred, predominantly in lower elevation, more arid ranges with fewer water sources (Epps *et al.*, 2004). The naturally occurring stratification in elevation, water source availability and forage quality (water content and digestible nitrogen) across these ranges coupled with limited intermountain movements creates an ideal study system to explore individual and population level differences in thermoregulatory patterns.

Here, we leveraged RIT biologging technology in wild and free-ranging desert bighorn to (i) detect and determine the frequency of year-round water use, (ii) evaluate sexual and population-level differences and seasonal patterns in body temperature variation and (iii) test whether body temperature variability predicted mortality risk. We hypothesized that water consumption would be reliably detected and would differ by season and range. We expected water consumption frequency to increase across all ranges in the hotter months and to be higher in lower elevation mountain ranges with poorer quality forage. We used daily body temperature metrics (minimum, maximum, median and amplitude) to assess thermoregulatory patterns in males and females across an elevational gradient of mountain ranges over the course of 1 year. We hypothesized that body temperature patterns would differ between sexes throughout the year. We predicted that males, with their larger body size, would exhibit less heterothermy than females especially during lambing, but that they may exhibit more heterothermy during the rut (mating season). We also predicted that individuals in lower, hotter mountain ranges would exhibit increased heterothermy than higher, cooler mountain ranges. Finally, we hypothesized that body temperature variation would be a reliable indicator of mortality risk. Specifically, we predicted that increased body temperature variation would be associated with increased mortality risk.

## Materials and methods

### Study area

**Figure 1:**
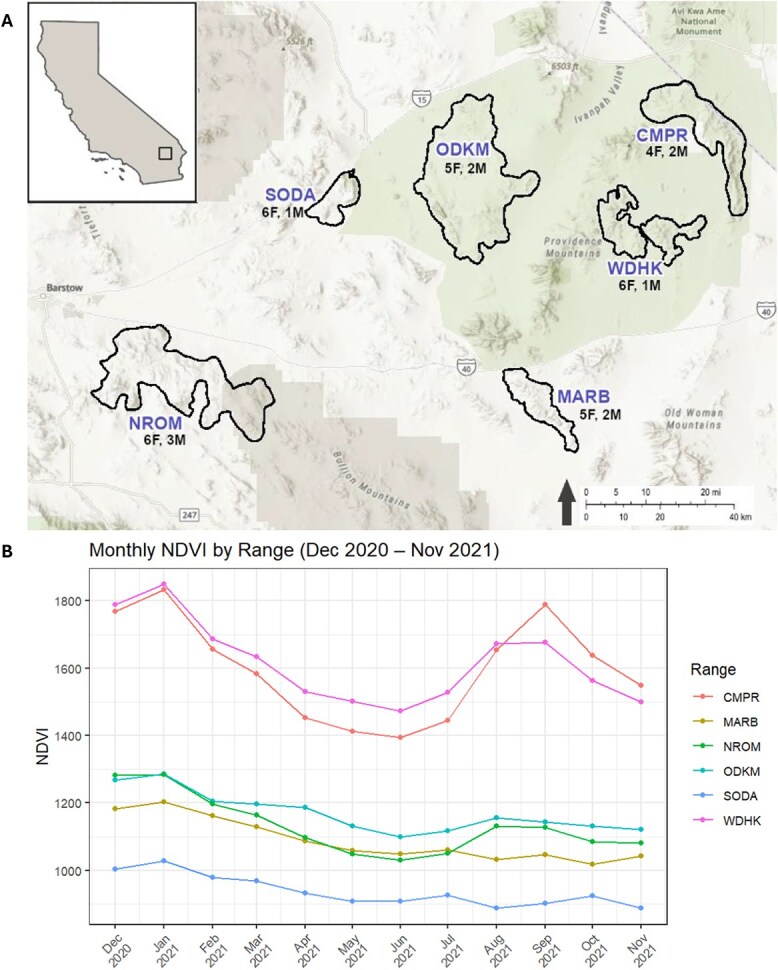
(**A**) RITs were deployed in 43 desert bighorn sheep (female 32, males 11) across six mountain ranges in the Mojave Desert, capturing broad variability in forage quality. Data were collected between November 2020 and December 2021, and the map shows the number of females (F) and males (M) captured per range. (**B**) The average monthly normalized daily vegetation index for each range during the study period, highlighting the variation in forage quality between ranges.

This study was conducted within and adjacent to the Mojave National Preserve in the central Mojave Desert of California, USA (Fig. 1A). This study focused on six desert bighorn populations located in the Newberry/Rodman/Ord (NROM), South Soda (SODA), Marble (MARB), Old Dad Peak (ODKM), Woods/Hackberry (WDHK) and Castle/Piute Mountains (CMPR). These populations, distributed across island-like mountain ranges, span from low, arid desert to more temperate basin-and-range. The SODA population has the lowest maximum elevation (738 m), followed by MARB (1163 m), ODKM (1498 m), CMPR (1769 m), WDKH (1885 m) and NROM (1918 m), with differences between minimum and maximum elevation ranging from 462 m (SODA) to 1366 m (NROM; USGS: TNM Download v2 nationalmap.gov, Table S1). Temperatures fluctuate from below freezing (−6°C) to extreme heat (50°C) depending on the mountain range and season. The Mojave Desert receives an annual average of 137 mm precipitation (ranging 34–310 mm), primarily between October and April, and some monsoonal moisture between July and September (Hereford *et al.*, 2006). Rainfall between October–November and January–February dictates 75% of the diet quality from February–June (Wehausen, 2005). Notably, across the southwestern USA, the year 2021 was recorded to be the 12th driest year in the last three centuries (Williams *et al.*, 2022) and the normalized difference vegetation index (NDVI; a measure of vegetation greenness, sometimes used as an index of diet quality in this system, e.g. Creech *et al.*, 2016) varied greatly across the six focal ranges during this period (Fig. 1B). Human activity was minimal across all six study sites during the study period except for an active gold mine in the northern portion of CMPR which may act to deter predators.

### Animal handling

For this study, the California Department of Fish and Wildlife coordinated the capture and handling of 49 desert bighorn (35 females, 14 males) from six focal mountain ranges in the central Mojave Desert during 3–12 November 2020 (Fig. 1A). Captures were conducted by a professional contractor, Leading Edge Aviation*,* using the helicopter net-gun method (Krausman *et al.*, 1985). Upon capture, bighorn were transported to a basecamp for processing in accordance with established animal handling protocols (Sikes, 2016). Animals were aged by assessing tooth replacement and counting horn annuli (Hemming, 1969). Every animal was fitted with a Vertex Plus Iridium (700 g; VECTRONIC Aerospace Inc.) global positioning (GPS) collar, a very high frequency collar (Telonics Inc.) and identifiable ear tags. Additionally, every individual received a RIT (100 g FIWI from VECTRONIC Aerospace Inc.), specifically designed for use in free-ranging ruminant animals to record internal body temperature and heartrate (Signer *et al.*, 2010). RITs were administered down the pharynx using an applicator and were retained in the reticulum for the duration of the study. Each animal was returned to its original capture location after all samples were collected, data were recorded and a photo was taken.

All capture and animal handling procedures were reviewed and approved by the California Department of Fish and Wildlife’s Wildlife Health Lab and the Oregon State University (IACUC-2019-0017) and the National Park Service (CA_MOJA_Beechler.Epps_DBHS_2020.A2) Institutional Animal Care and Use Committees. Research was permitted by the National Park Service (MOJA-2020-SCI-0031) and the Bureau of Land Management (DOI-BLM-CA-D010-2018-0002-EA).

### GPS collar and RIT programming

GPS collars were programmed to record the individual’s location every 2 hours and upload locations to a website once daily. Mortality alerts were issued via email after 8 hours without movement. GPS collars also communicated with RITs that were placed during capture. Each RIT was programmed to record reticulum temperature (hereafter referred to as body temperature, Vesterdorf *et al.*, 2022) every 3 min. These data were transmitted to and stored on the GPS collars, which were programmed to drop off after 12 months of deployment, in December 2021. Eleven animals (10 females, 1 male) died during the study; however, only 8 of female mortalities had usable RIT data (Fig. S2). One ram was re-captured in November 2021 to recover a GPS collar with a known communication failure. Once the collars were recovered, RIT data sets were downloaded to a computer.

### RIT temperature correction

Each RIT was calibrated prior to shipping by the manufacturer, VECTRONIC Aerospace Inc., using a water bath tempered to 37–38°C. RIT temperature readings were validated against a Greisinger thermometer GTH175/Pt with a PT1000 sensor and consistently recorded water bath temperatures higher than the thermometer. The manufacturer provided a correction factor ranging from 0.1 to 0.4°C for each RIT, which we subtracted from all raw body temperatures prior to analysis.

### Identifying and validating drinking events

**Figure 2:**
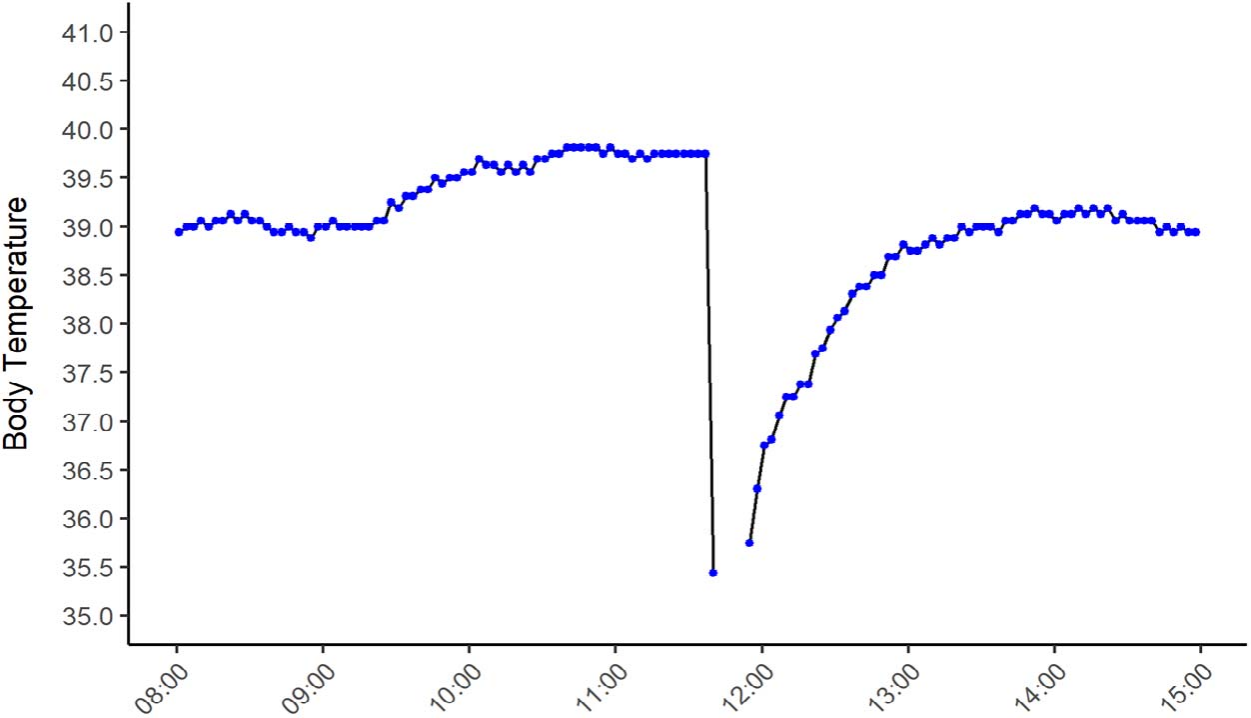
An example of the change in body temperature (°C) before, during and after consumption of surface water by a female desert bighorn sheep with an RIT. The pronounced drop of the drinking event is around 11:45, followed by a gradual return to core body temperature around 13:30.

We developed protocols to identify drinking events and validated them using camera data at water sources (see Appendix S1). These drinking events were used to describe bighorn water use patterns as described below but were removed from the dataset prior to calculating body temperature metrics (creating a dataset called corrected RIT temperature used below). This ensured that the pronounced drop in body temperature during a drinking event and gradual temperature increase post-drinking (Fig. 2) did not influence the body temperature metrics used to describe heterothermy.

### Water use by a desert ungulate

We characterized water use as the rate of visitation per calendar month across six Mojave Desert ranges from December 2020 to November 2021. Daily drinking was recorded as a binary event (0 = no visit; 1 = ≥1 visit) based on the RIT-determined drinking events. We then collapsed daily records to an individual × month dataset, computing the number of drinking days and days observed for each collared bighorn during each month with RIT data. We fit a binomial mixed-effects model (logit link) with the number of drinking days relative to the days observed each month as the response variable, fixed effects included month (YearMonth) and population (Range), their interaction and a random intercept for individual (BHS_ID) to account for repeated measures. Models were estimated by maximum likelihood (Laplace approximation) using the *lme4* package (Bates *et al.*, 2015) and model predictions were generated using the *emmeans* package (Lenth and Piaskowski, 2025) in R version 4.4.2 (R Core Team, 2024). From the fitted model we obtained estimated marginal means on the response scale for each Range × Month combination and expressed them as expected drinking days per calendar month by multiplying the predicted daily probability by the number of days in that month; 95% Wald confidence intervals were transformed identically.

### Body temperature and model metrics

Multiple metrics can be used to assess body temperature and to track the 24-h circadian rhythm. We calculated daily values for each individual of minimum, maximum and median using the corrected RIT temperature data to describe overall trends and to detect periods of hypo- and hyperthermia that may be driving temperature variation. Amplitude, interquartile range (IQR) and heterothermy index (HI) are each slightly different metrics to describe body temperature variation. For our study we tested all three metrics of body temperature variation (Appendix S2); due to similar results we focus on amplitude in our main analyses. Amplitude was calculated by subtracting the daily minimum from the maximum.

To account for the effect of body size on thermoregulation and to address the potential for weight to fluctuate from values recorded at capture, we calculated a scaled body mass index (BMIsc, Peig and Green, 2009) for each individual: ${\hat{M}}_i={M}_i{\left[\frac{L_0}{L_i}\right]}^{b_{\mathrm{SMA}}}$. In this equation, the predicted body mass (${\hat{M}}_i$) for an individual *i* equals the individual body mass (${M}_i$) times the mean length of all individuals in the study (${L}_0$) divided by the individual body length (${L}_i$) raised to the scaling exponent (${b}_{\mathrm{SMA}}$) estimated by the standardized major axis (SMA) regression of *M* on *L*. Age was categorized into three groups (1–3): yearling (1–2 years; 3 females, 3 males), young adult (2–5 years; 11 females, 6 males) and mature adult (6+ years; 18 females, 2 males). To mitigate potential capture effects, account for varying collar-drop dates and to allow for seasonal analysis without confounding inter-year variation we limited analyses to temperature data collected 1 December 2020 through 30 November 2021. Data were also filtered to the day prior to a mortality or collar drop event. Summary statistics by sex were calculated using the median value of each body temperature metric.

### Drivers of body temperature variation

We analysed the four daily thermal metrics—median, minimum, maximum and amplitude—with separate linear mixed-effects models (LME). Fixed effects reflected *a priori* biological hypotheses and included sex, age class, calendar month (YearMonth), mountain range and BMIsc. To test for sex-specific thermoregulatory patterns across space and season, we included Sex × Range and Sex × YearMonth interactions. The Sex × Age category was evaluated using drop-one likelihood ratio tests (LRTs) across all six response variables and found to contribute no explanatory power in any model (all *χ*^2^(2) ≤ 3.58, all *P* ≥ 0.16); it was therefore removed from all final models. Age class was retained as a main effect given its *a priori* biological relevance. BMIsc was mean-centered and entered as a quadratic term to allow nonlinear size effects while minimizing collinearity and keeping the intercept interpretable at mean BMI (Schielzeth, 2010). Factors were re-levelled so the intercept represents a prime-age adult (age class 2; 2–5 years) female in the ODKM during December 2020 at mean BMIsc.

Because daily body temperature series within animals are temporally ordered, we modelled within-individual autocorrelation using continuous-time AR(1) structure (corCAR1) on the residuals, indexed by a per-animal day counter (day-index) to accommodate occasional missing days (Pinheiro *et al.*, 2025). Mixed models were fit using restricted maximum likelihood with a random intercept for individual (1|BHS_ID) and correlation = corCAR1 (~day_index|BHS_ID) using the *lme* function in the *nlme* package (Pinheiro *et al.*, 2025). The CAR(1) autocorrelation structure was strongly supported over models with no autocorrelation term across all six response variables (ΔAICc = 144.7–4444.9; all LRT *P* < 0.0001). Autocorrelation reduction was confirmed by (i) normalized-residual autocorrelation functions computed per animal, showing marked shrinkage of lag-1 correlations towards zero, and (ii) Ljung–Box tests across animals showing reduced residual autocorrelation at lag 1 following CAR(1) correction; however, some medium-range autocorrelation persisted at lags 7–14 in a subset of individuals, which would inflate Type 1 error conservatively. Model fit was evaluated using marginal *R*^2^ (fixed effects only) and conditional *R*^2^ (fixed + random effects; Nakagawa and Schielzeth, 2013, implemented via the *performance* package; Lüdecke *et al.*, 2021). Model assumptions were evaluated by inspecting normalized residual Q-Q plots and residuals-versus-fitted plots; residuals showed mild right skew in some metrics (skewness range: −0.29 to 1.08) but flat residuals-versus-fitted relationships across all models, and fixed-effect inference was considered robust given *n* = 14 006 observations.

### Association between heterothermy and survival

Variation in body temperature metrics was evaluated as a predictor of mortality risk using survival data from 43 individuals monitored between November 2020 and December 2021, during which 8 mortality events occurred (Fig. S2). The survival analysis was conducted in R version 4.4.2 (R Core Team, 2024). General data manipulation was conducted with *dplyr* package (Wickham *et al.*, 2023). Data summaries were generated with the *textreg* package (Leifeld, 2013) and plots were generated with *ggplot* (Wickham, 2016) and *patchwork* (Pedersen, 2024) packages.

The survival data set was prepared for analysis by implementing a custom interval-based aggregation function that partitioned time-series data into fixed-width intervals of 100 days for each individual. We used an interval-based survival structure to accommodate time-varying covariates and capture dynamic changes in the body temperature metrics (daily minimum, maximum, median and amplitude), allowing for more accurate modelling of survival risks across repeated observations within individuals. A fixed interval length of 100 days was selected to balance temporal resolution, model stability, and the sparse distribution of events (*n* = 8 deaths, subjects = 43, sample size = 165), ensuring intervals were short enough to preserve adequate resolution in the body temperature metrics while long enough to allow for the sparsity of events.

The custom aggregation function grouped observations by subject ID and calculated interval-specific start and stop times, event status, and mean values for the body temperature metrics within each 100-day interval. Static variables were retained using the first observed values in each interval. The resulting dataset contained one row per subject per interval, structured for time-to-event modelling. Survival time and censoring status were encoded using a right-censored survival object created with the *surv* function from the *survival* package (Therneau, 2024a).

We fitted five separate Cox proportional hazard mixed-effects models (one per body temperature metric: minimum, maximum, median, amplitude and an age-only base model) using *coxme* function from the *coxme* package (Therneau, 2024b), following an iterative approach to avoid multicollinearity among the highly correlated thermal metrics in which each body temperature metric was included separately alongside animal age. This strategy controlled for multicollinearity among the body temperature metrics, which were highly correlated (Fig. S3). Three additional models tested the body temperature variation metrics HI, IQR and amplitude against the same age-only base model. Each model included centered age (mean = 4.8 years) as a fixed effect alongside the body temperature metric of interest. All models included random intercepts for individual identity (BHS_ID) and mountain range (Range) to account for repeated measures and population-level clustering. The significance of each body temperature was assessed by LRT comparing each metric model to the age-only base model. Only one male mortality occurred during the study period, precluding formal evaluation of sex-specific mortality risk. The proportional hazards assumption was evaluated using scaled Schoenfeld residuals via the *cox.zph* function in the *survival* package (Therneau, 2024a) with no significant violations detected (all *P*-value >0.05).

To visualize the effect of age on survival, we used a custom plotting function that wraps around the *ggemmeans* function from the *ggeffects* package (Lüdecke, 2018), to extract marginal predictions and generate partial effects plots. Prediction series were generated across the observed values for each focal covariate showing the predicted probability of mortality while all other fixed-effect variables were held constant at their typical values. Random effects were excluded from the predictions. As such, the plots reflect population-level estimates based solely on the fixed effects. Shaded ribbons represent 95% confidence intervals derived from the model’s fixed-effect estimates.

## Results

### Study population and body temperature metrics

RIT data were retrieved from 49 individuals (35 females, 14 males; 7 595 214 raw 3-min observations). RIT temperature data were transmitted to the Vectronics GPS collar every 3 min, yielding up to 480 transmissions per animal per day. The GPS collars successfully received at least a timestamp on 99.7% of all possible 3-min transmission intervals, indicating near-continuous collar-RIT communication. Of the 49 desert bighorn captured for this study, six individuals were removed due to capture related mortality or equipment failure within the first 2 months, leaving 43 individuals (32 females, 11 males; Fig. 1A) and 7 242 599 3-min observations available for analyses. Of these, 92.7% contained a valid body temperature reading (SD = 7.0% across individuals; median = 96.2% across animal-days); the remaining 7.3% recorded a timestamp but no temperature value. Following the identification and removal of drinking events, 6 592 962 3-min temperature observations (91.0% of valid readings) were available for body temperature analyses and summarized into 14 006 daily observations.

Using the 14 006 daily observations we calculated the median value for each metric (Table 1). Notably, in our effort to validate whether drinking events observed on camera were reflected in the RIT data, we observed a RIT drinking event associated with every instance of an animal photographed at a water source among the 50 photos examined across all ranges and sexes. While no false negatives were detected across 50 camera-validated comparisons, the false positive rate cannot be precisely estimated because desert bighorn also drink from ephemeral water sources such as tinajas that were not monitored by cameras; cross-referencing RIT-detected events at unknown locations with GPS data and local precipitation records provided supportive but not definitive evidence of drinking at these sites. The dataset used for assessing the effect of season and range on the probability of drinking included 9731 days with no drinking events and 4275 days with drinking events.

**Table 1:** Summary of daily body temperature statistics for 43 desert bighorn (32 females, 11 males) between December 2020 and November 2021

**Daily body temperature metrics**	**Female**	**Male**
Minimum	38.01 ± 0.36°C	37.98 ± 0.33°C
Maximum	39.70 ± 0.40°C	39.55 ± 0.38°C
Median	38.89 ± 0.21°C	38.80 ± 0.21°C
Amplitude	1.75 ± 0.50°C	1.62 ± 0.48°C
IQR	0.57 ± 0.24°C	0.56 ± 0.22°C
HI	0.47 ± 0.18°C	0.45 ± 0.17°C

These values were calculated using the median daily value and standard deviation for each metric and do not account for model covariates.

### Water use by a desert ungulate

**Figure 3:**
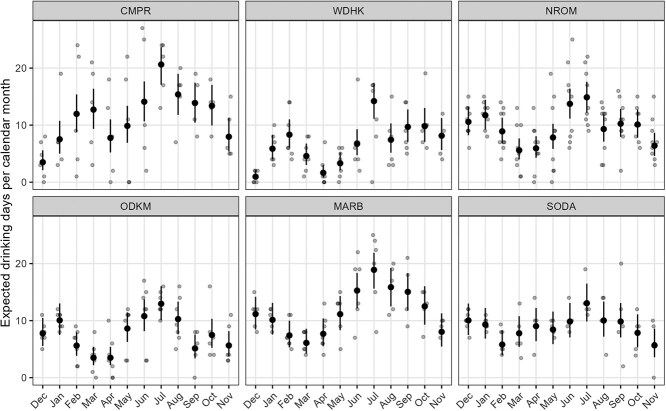
Expected number of days that desert bighorn sheep drank surface water per calendar month (December 2020–November 2021) for six populations in the Mojave Desert of California. Grey points show observed individual-month counts of days with ≥1 drinking event (scaled to days per month). Black points are marginal means; vertical bars are 95% Wald confidence intervals. ‘Expected’ values are back-transformed to days for each month.

All focal animals in all six desert bighorn study populations used water intermittently year-round, and the frequency of visits differed among mountain ranges and across the year (Fig. 3, Table S2). In mid-winter (December), desert bighorn in two of the higher elevation ranges visited water the least—individuals visited water and drank, on average, at least once per month in the WDHK and 3–4 days a month in CMPR. Conversely, in the lower elevation ranges of SODA and MARB desert bighorn maintained moderate water use of roughly 10–11 days per month, this was similar rate as the higher elevation range of NROM, and the mid-elevation range of ODKM averaged 8 days a month. By mid-summer (July), all populations increased their use of water, but the amplitude of that seasonal rise differed by range: CMPR showed the most pronounced increase, with individuals drinking water on average 21 days per month; MARB rose to 19 days per month; WDHK, NROM, ODKM and SODA clustered between 13–15 days per month. Thus, the hotter, drier, low elevation populations drank regularly year-round with a modest increase in the summer, while the cooler, wetter, high-elevation populations shifted from sporadic winter use to frequent summer use. Despite the inter-population differences, intra-population individual heterogeneity was modest (random-intercept SD ≈ 0.40 on the logit scale), shifting an individual’s expected monthly count by only a few days around its population mean.

### Drivers of body temperature variation

Seasonal variation (YearMonth) was the dominant driver of body-temperature dynamics across all LME (Table S3a). Daily maximum body temperature and diel amplitude rose steadily from winter to late spring and early summer, whereas daily minima were lowest in early spring. Specifically, maximum body temperature peaked May through August and amplitude peaked from February through August (LME ‘maximum’: *β* = 0.154–0.343, *P* < 0.001); ‘amplitude’: *β* = 0.364–0.553, *P* < 0.001). Minimum body temperature reached its lowest values February through May (LME ‘minimum’: *β* = −0.233 to −0.367, *P* < 0.001). Thus, body temperature variation was elevated for two physiologically distinct reasons—in the spring because minima were especially low, and in the summer because maxima were especially high. HI and IQR showed the same seasonal rise (Table S3b).

Animals in the hottest, driest ranges showed greater body temperature variation (Table S3a). Amplitude was higher in SODA and MARB relative to ODKM (LME ‘amplitude’: SODA *β* = 0.367, *t* = 2.618, *P* = 0.014; MARB *β* = 0.491, *t* = 3.675, *P* = 0.001). The drivers of body temperature variation differed by range: maxima were higher in both SODA and MARB relative to ODKM (LME ‘maximum’: SODA *β* = 0.268, *t* = 2.707, *P* = 0.012; MARB *β* = 0.211, *t* = 2.242, *P* = 0.033), while minima were lower only in MARB (LME ‘minimum’: *β* = − 0.280, *t* = −2.458, *P* = 0.021), suggesting distinct thermoregulatory constraints across environments.

**Figure 4:**
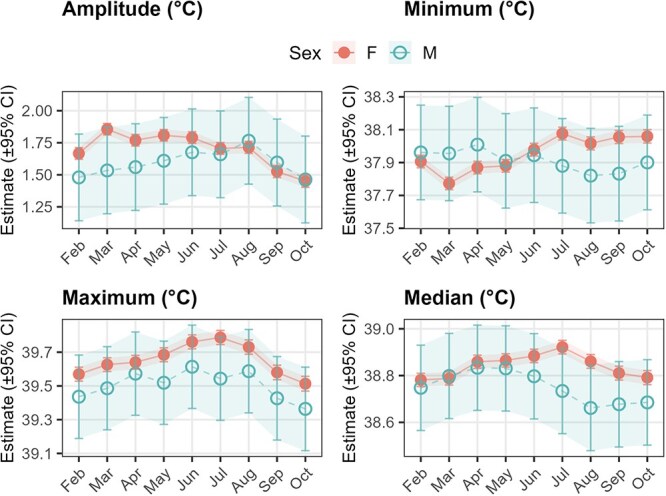
Sex-specific seasonal patterns in daily body temperature metrics for desert bighorn sheep across six Mojave Desert populations from February to October 2021. Points show monthly marginal means ± 95% confidence intervals from LME fit separately for each metric (amplitude, minimum, maximum and median body temperature in degree Celsius), averaged over ranges, age classes and BMIsc. Connecting lines are shown as a visual guide and do not imply a continuous seasonal effect; month was modelled as a categorical predictor with December 2020 as the reference. The figure highlights two ecologically distinct seasonal routes to greater daily body temperature variation in females: lower daily minima during the spring lambing season (February–May) and higher daily maxima during peak summer heat (June–July). Males showed lower body temperatures relative to females in late summer through early autumn (July–September), coinciding with the mating season, without a corresponding increase in daily temperature variation.

Sex had no overall main effect on any daily body temperature metric (all LME: |*t*| ≤ 0.739, *P* ≥ 0.474 for sex = male; Table S3a). Body size (BMIsc) had no linear or quadratic effects across the models (all LMEs: |*t*| ≤ 1.81, *P* ≥ 0.083). Young animals (Age.Cat1; ≤2 years) had significantly higher daily median, maximum, and minimum body temperatures than prime-aged adults (LME median *β* = 0.135, *t* = 2.229, *P* = 0.034; maximum: *β* = 0.185, *t* = 2.283, *P* = 0.031; minimum: *β* = 0.216, *t* = 2.198, *P* = 0.037). Mature adults (Age.Cat3; ≥6 years) did not differ significantly from prime-age adults in any metric (all *P* ≥ 0.32). However, body temperature dynamics showed two seasonally distinct sex-driven patterns across the year (Fig. 4, Table S3a).

First from late winter into spring, coinciding with the lambing season, females displayed significantly greater body temperature variation than males, driven predominately by lower daily minima. Specifically, Sex × YearMonth interactions were negative from February through June for amplitude (LME ‘amplitude’: *β* ≈ −0.324 to −0.117, *t* = −7.395 to −2.631, all *P* < 0.009), while daily minima were significantly higher in males than females from February through May (LME ‘minimum’: *β* ≈ 0.117 to 0.270, *t* = 3.148–7.267, all *P* ≤ 0.002). Secondly, females had significantly higher maximum body temperatures in July (LME ‘maximum’: July; *β* = −0.157, *t* = −3.894, *P* < 0.001). Conversely, in late summer to early autumn (July–September) males daily minima and median temperatures fell relative to females (LME ‘minimum’: *β* ≈ −0.136 to −0.109; *t* = −3.596 to −2.912, all *P* ≤ 0.004; ‘median’: *β* ≈ −0.148 to −0.082; *t* = −5.287 to −2.902, all *P* ≤ 0.004), overlapping with the rut. Notably, the lower minima and median temperatures in the males during this period did not translate into higher daily body temperature variation—Sex × YearMonth terms for amplitude were non-significant for males from July through November (all *P* ≥ 0.278). Together, these results highlight that females experienced greater body temperature variation than males by dual mechanisms: lower daily minima in the spring and elevated daily maxima during the hottest months, while males reduced their body temperature during the summer mating season without increased heterothermy.

### Association between heterothermy and survival

**Figure 5:**
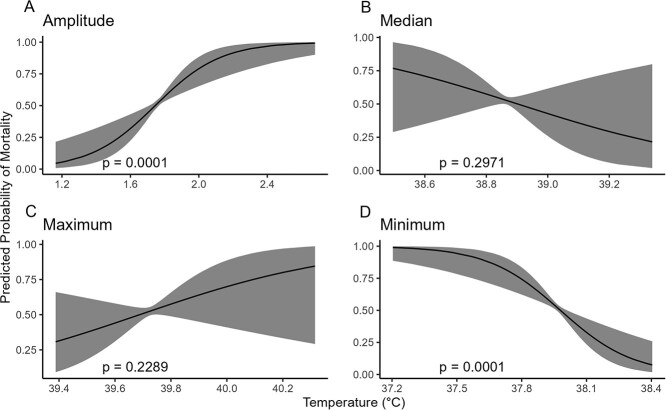
Partial effects plots exhibiting the predicted probabilities of mortality of desert bighorn sheep (mortality event = 1) for each body temperature metric tracked from November 2020 to December 2021. Plot **A** shows the significant relationship between increased amplitude (heterothermy) and increased risk of mortality. Plot **B** suggests a slightly negative, though non-significant trend between lower daily median body temperature and increased risk of mortality. Plot **C** shows a slightly positive, though non-significant relationship between daily maximum body temperature and mortality. Plot **D** shows the strong significant relationship between lower daily minimum and increased mortality risk. Predictions were generated using the observed values for each focal covariate while all other fixed-effect variables were held constant. Shading represents 95% confidence intervals.

Desert bighorn that died exhibited lower daily minimum body temperatures and greater daily body temperature variation compared to survivors during the corresponding 100-day observation windows (Fig. 5, Table S4a). Age was not significantly associated with mortality risk as a standalone predictor (*β* = 0.262, SE = 0.157, LRT *χ*^2^(1) = 2.95, *P* = 0.086) and its effect remained non-significant across all body temperature metrics. Minimum body temperature was significantly associated with mortality risk across all individuals (*β* = −5.926, SE = 1.700, LRT *χ*^2^(1) = 14.83, *P* < 0.001), per 0.1°C decrease in mean daily minimum temperature, the hazard of mortality increased by ~81% (HR = exp(−5.926 × −0.1) ≈ 1.81; *P* < 0.001). Mean daily amplitude was strongly associated with increased mortality risk across all individuals (*β* = 5.215, SE = 1.481, LRT *χ*^2^(1) = 15.81, *P* < 0.001), per 0.1°C increase in amplitude, the hazard of mortality increased by ~68% (HR = exp(5.215 × 0.1) = 1.68; *P* < 0.001). Neither median body temperature (*β* = −2.956, SE = 2.883, LRT *χ*^2^(1) = 1.09, *P* = 0.297) nor maximum body temperature (*β* = 2.710, SE = 2.233, LRT *χ*^2^(1) = 1.45, *P* = 0.229) was significantly associated with mortality risk. Taken together, these data indicate that mortality risk was driven specifically by a drop in daily body temperature minima resulting in greater heterothermy, rather than by elevated maxima or shifts in overall temperature level.

Across all individuals, greater body temperature variation was significantly associated with increased mortality hazard (Fig. S1, Table S3b) whether quantified as the HI (*β* = 14.807, SE = 4.143, LRT *χ*^2^(1) = 14.27, *P* < 0.001), IQR (*β* = 12.908, SE = 3.910, LRT *χ*^2^(1) = 13.36, *P* < 0.001), or amplitude (LRT *χ*^2^(1) = 15.81, *P* < 0.001). The consistency of this result across metrics confirms that the association reflects a genuine physiological signal rather than an artefact of any single measure.

## Discussion

Our study used RITs in a free-ranging desert ungulate to reveal year-round drinking behaviour and sex-based differences in seasonal thermoregulatory patterns. In this drought year, desert bighorn in the lower elevation ranges relied on consistent year-round water use, while individuals in high elevation ranges showed pronounced increases in water use during the hot summer months. Strikingly, we found that individuals exhibiting greater body temperature variation had lower survival. Individuals that were chronically unable to maintain their body temperature minima, resulting in greater long-term heterothermy, were more likely to die than other individuals during the same observation windows. Females across ranges exhibited two distinct seasonal patterns of heterothermy during spring lambing and hot summer months, whereas males lowered their body temperature during the hottest months and mating season.

Contrary to the literature (Gedir *et al.*, 2016; Harris *et al.*, 2020), desert bighorn across all six populations used surface water year-round, with individuals in the lower elevation ranges maintaining constant use throughout the year and the higher elevation ranges experiencing more pronounced summer increases. A previous study in this area by Glass *et al.* (2022) used a combination of GPS data and cameras at water sources to assess visitation rates in four mountain ranges including MARB and CMPR between May and August 2019—both ranges experienced notably more precipitation (~19 and 39 mm, respectively) in April and May than in 2021. Their results showed minimal water use by bighorn in CMPR in May and June and consistently higher use by animals in MARB across all 4 months (Glass *et al.*, 2022). Similar to other studies (Cain *et al.*, 2008a, 2008b; Gedir *et al.*, 2016), Glass *et al.* (2022) suggested that animals likely meet their water requirements through the forage they consume during the non-summer months and are able to prolong this in the higher elevation ranges. However, many of these studies relied on animals coming into artificial or known water sources to access water and often did not include the winter months in their analyses.

Biologgers, however, allowed us to obtain high resolution drinking data for this desert ungulate and better understand how reliant individual bighorn are on water year-round, particularly in the lower elevation ranges with poorer quality forage (Epps *et al.*, 2004). Unlike camera- or GPS-based approaches that require animals to visit known water sources, RITs can detect drinking events at ephemeral sources such as tinajas that may fill only briefly following precipitation events, providing a more complete picture of true water intake across the landscape. Specifically, RITs were able to detect drinking events that did not occur at known water sources, such as events at tinajas (rock basins) that may intermittently fill with water after rainfall. There was a pronounced drop in drinking rates in March and April, trailing a January peak NDVI (Fig. 1B). This delayed decline in drinking rates may be due to individuals getting the water they needed from non-green forage, such as brittlebrush (*Encelia farinosa*) which have yellow flowers not captured by NDVI. This may also reflect females staying closer to escape terrain with young lambs to reduce the risk of predators at water sources (Harris *et al.*, 2020).

Our results raise the questions: are some bighorn populations behaviourally adapted to surface water sources more than others or are some water sources simply more accessible? For example, animals in the CMPR appear to have a disproportionate affinity for using surface water given the increased elevation and higher quality forage, but most of these animals are also located near a mine that provides multiple wildlife water developments and may reduce predator pressure in the area, consistent with the ‘landscape of fear’ framework (Laundre *et al.*, 2010). Further research into the behaviour of surface water use in the context of diet quality and other activities may help elucidate these differences. Importantly, this study took place during the megadrought year of 2020–21 (Williams *et al.*, 2022) and offers valuable insight into the importance of year-round water availability during drought periods when forage quality is reduced; however, as this represents a single drought year, long-term monitoring across years with varying precipitation regimes will be needed to determine whether these patterns are consistent. It is critical for wildlife managers to consider this when managing water sources, as year-round water sources will likely continue to be necessary for the lower elevation populations already facing higher rates of extinction (Epps *et al.*, 2004), and individuals in higher elevation ranges may have greater reliance on summer water sources as global temperatures continue to rise.

Seasonality differentially affected the thermoregulation patterns of male and female desert bighorn, in contrast to a study on free-ranging Arabian oryx (Streicher *et al.*, 2017) that found that body temperature variation did not differ between sexes. After adjusting for range, season, age and body size, females exhibited significantly greater heterothermy during the peak-lambing season (February–May) and the hot summer months (May–July). Young animals (≤2 years) also showed higher daily median, maximum and minimum body temperatures than prime-age adults across all models, though this effect did not interact with sex or season. Body temperature variation can be influenced by periods of hypothermia, resulting in lower daily minima, or hyperthermia, resulting in higher daily maxima. During the lambing season, female desert bighorn exhibited hypothermia. Conversely, during the summer, they exhibited hyperthermia. A study on vervet monkeys similarly found that females exhibited hypothermia during gestation, potentially to reduce foetal hyperthermia, but then experienced hyperthermia and greater heterothermy during lactation and a reduction in available resources (McFarland *et al.*, 2024). For desert bighorn, the lower daily minimum body temperatures may also help to reduce foetal hyperthermia and also be the result of the high energetic demands of pregnancy and lactation (Denryter *et al.*, 2021). The period of hyperthermia in the early summer coincides with a steep decline in forage quality (Wehausen, 2005), lactation and summer heat waves. The latter may be the driving force of higher daily maximum temperatures if females are not able to physically or behaviourally thermoregulate enough to minimize heat load (Cain *et al.*, 2006, 2008a). Another possibility is that females are thermally flexible and utilize adaptive heterothermy to elevate their body temperature through the reduction of evaporation, thereby conserving water (Mitchell *et al.*, 2002). However, whether females are actively allowing this rise through adaptive thermoregulation or are limited in their capacity for evaporative cooling under peak heat loads remains unclear—distinguishing these mechanisms would require additional data on water balance and behavioural thermoregulation (Hetem *et al.*, 2016). While the absence of a significant association between elevated daily maxima and mortality risk in our data suggests that this degree of hyperthermia may be tolerable under conditions observed, sustained or more extreme hyperthermia would ultimately become fatal (Angilletta *et al.*, 2010).

While males did not exhibit more body temperature variation during the summer mating season (rut), it was notable that they had significantly lower daily minima and median values than females from July through September (Table S3a). This apparent downregulation of body temperature clearly was not related to body size (BMIsc), but may be biologically driven to reduce testicular hyperthermia (Skinner and Louw, 1966; Rizzoto and Kastelic, 2020), a strategy also documented in male dromedary camels during rut (Grigg *et al.*, 2009). Other studies have found that heterothermy is not dictated by body size alone but by a range of factors including energetic stores, water availability, behaviour or micro-habitat use (Hetem *et al.*, 2016). Further research to assess if males are selecting for cooler micro-climates or are less active during the hottest part of the day than females may help provide insights on how they are able to maintain lower body temperature during the hottest part of the year and during the rut. It is possible these differences may vary by sexual maturity but the smaller male sample size limited our ability to evaluate sex-specific interactions with age and thermoregulation.

Sustained heterothermy is broadly accepted as being an indicator of energetic trade-offs in endothermic mammals (Boyles *et al.*, 2011b; Hetem *et al.*, 2016; Morales *et al.*, 2021) and is associated with reduced reproductive fitness (Maloney *et al.*, 2017) and poor body condition as a result of disease, starvation and dehydration (Hetem *et al.*, 2016; Botha *et al.*, 2023). However, very few studies have explored the association between long-term heterothermy and survival (Dammhahn *et al.*, 2017). Our survival analysis strongly suggests that animals that died had different thermal profiles than those that were alive during the same corresponding period (Table S4a). The significantly higher body temperature variation metrics and lower daily minima in animals that died are notable and consistent with other studies suggesting that greater body temperature fluctuations are often indicative of physiological stress, nutritional deficits, or reduced fitness (Hetem *et al.*, 2016; Boyers *et al.*, 2019 ) or predation (Morales *et al.*, 2021). Specifically, declines in daily temperature minima drove this association, indicating that mortalities likely occurred due to energetic constraints as opposed to overheating. This association between hypothermia and mortality risk is particularly striking during a megadrought year (Williams *et al.*, 2022) when one might expect high ambient temperatures to result in hyperthermia and increased mortality risk (Calleja-Agius *et al.*, 2021). The heterothermy–mortality association was consistent across all three body temperature variation metrics examined—amplitude, HI and IQR—and held across all individuals in our sample regardless of sex or range affiliation (Table S4b), indicating a genuine physiological signal rather than a population- or sex-specific phenomenon. Chronically reduced daily temperature minima, resulting in greater amplitude of the daily thermal cycle, thus serve as an early physiological signal of energetic stress that preceded death in this population—a pattern also documented in aardvarks (Rey *et al.*, 2017). Although real-time data are still challenging to obtain from free-ranging animals, future technological advances could provide useful insights for both wildlife and livestock managers. Whether males exhibit similar heterothermy-associated mortality patterns represents an important avenue of future research.

Our study provides novel insights into the year-round use of surface water by desert ungulates especially in lower elevation areas with poorer quality forage. However, it also raises questions as to whether other constraints influence surface water use and limit its benefits to animals and their resilience to drought. Furthermore, it appears that energetic restrictions in the springtime drive greater heterothermy more than summertime heat waves. Although greater heterothermy was associated with lower survival, mortalities were well distributed across the study period. Alarmingly with respect to conservation, given that high adult female survival is key to population stability for long-lived ungulates (Gaillard *et al.*, 1998), females appear more sensitive to increased energetic demands, although they may also be more thermally flexible during extreme heat. Biologging allowed us to build the foundation to understand the mechanisms linking environmental change to fitness outcomes and evaluate resilience to environmental change. Given the increasing frequency of extreme heat events and prolonged droughts associated with climate change (Williams *et al.*, 2020), future studies should investigate the mechanisms driving individual variation in thermoregulation and the extent to which animals can behaviourally or physiologically compensate for environmental stressors. Additionally, integrating RIT-derived temperature data with environmental and behavioural datasets may offer deeper insights into how habitat use, foraging behaviour, and water reliance influence thermoregulation and survival. Ultimately, our findings establish continuous body temperature monitoring as a window into the physiological state of wild desert ungulates, capable of revealing not only how animals respond to seasonal and environmental stress but also which individuals are most at risk of dying. Even where data retrieval is not instantaneous, the thermal signatures identified here inform conservation managers about which populations, seasons, and environmental conditions generate the greatest physiological risk—knowledge that can guide proactive management of water sources, habitat and monitoring efforts in an era of intensifying drought.

## Supplementary Material

Web_Material_coag056

## Data Availability

The data underlying this article and the supplementary material will be shared on reasonable request to the corresponding author.
