# Supplementary material for: Biologging of body temperature in a desert ungulate reveals water use, sex-specific seasonal thermoregulation and heterothermy-associated mortality
Source: Conserv Physiol. 2026 Aug 6;14(1):coag056. doi: 10.1093/conphys/coag056 (PMC13446989; doi:10.1093/conphys/coag056)
Supplement: Supplementary file 1 [file web_material_coag056.zip › BiologgingDesertBighorn_SupplementaryMaterials_Prentice_Final_Revised_20260718_PROOFED.docx]

Supplemental Information

**Appendix S1. Validating the use of body temperature data for identification of drinking events.**

Unlike other core body temperature devices such as vaginal implant transmitters and subdermal transmitters, reticulum implant transmitters (RITs) exhibit fluctuations in reticulum temperature readings when ruminant animals drink water, due to water influx into the reticulum (Herberg *et al.*, 2018a; Vesterdorf *et al.*, 2022). Vesterdorf et al. (2022), in a climate-controlled study with captive domestic sheep, found that water consumption was generally characterized by a pronounced drop in RIT temperature, followed by a logarithmic increase until the temperature stabilizes, approximately 96 minutes post-drinking (Figure 2). Despite that strong short-term response, that study and another on domestic cattle (Beatty *et al.*, 2008) concluded that drinking events did not appear to affect the average daily relationship between reticulum and core temperatures. Studies on free-ranging moose, however, elected to remove RIT data associated with drinking events prior to analysis (Herberg *et al.*, 2018b; Thompson *et al.*, 2021).

We used rates of body temperature change between consecutive temperature readings (∆T) to identify water consumption (drinking events). Because water consumption is occasionally associated with missing data (NA values), we filled NAs with the previous known body temperature value prior to calculating ∆T. Vesterdorf et al. (2022) defined drinking events as any temperature recorded below the 95% lower limit for ∆T (- 0.4°C) and included the subsequent 96 minutes to allow for post-drinking temperature stabilization. Similarly, we calculated the lower limit of the 95% data threshold (- 0.36°C), rounded to - 0.4°C, and used that value for the ∆T cut off. To examine temperature stabilization after identified events, we calculated body temperature variance for each time point using a rolling window of 32 observations, or 96 minutes.

We observed that the stabilization period – a period of elevated body temperature variance following identified drinking events – varied widely and did not always occur within a 96-minute window. Therefore, we adjusted the period used to define a drinking event by using a combination of the ∆T threshold and a rolling variance. Specifically, a drinking event was initiated by a ∆T exceeding - 0.4°C coupled with a variance surpassing the median variance. The drinking event was terminated once the variance reverted below the median. These drinking events were then validated using camera data.

To test the sensitivity of RITs in identifying drinking events, we placed motion-activated trail cameras (Bushnell Trophy Cam) at opportunistic wildlife water developments from June-September in four of the six study areas Newberry/Rodman/Ord (NROM), Marble (MARB), Old Dad Peak (ODKM), and Castle/Piute Mountains (CMPR). Cameras were placed within 20 feet of the water sources and programmed to take one photo every ten seconds upon motion activation. We sorted the photos to identify when study animals were at the water sources. We then randomly selected 50 photos across the four study areas and checked if there was an associated RIT drinking event with the individual identified at the water source. Notably, this method of validation can only confirm true positive events – events where collar and RIT-monitored animals drank from a monitored water source. We have no means to estimate how often drinking events were inferred from temperature data but did not truly occur (false positives) because known water sources are not the only free-standing water available for bighorn to drink. The closest we could come to confirming drinking events at unknown water sources was by choosing several RIT drinking events that were not captured on camera, confirming the individual’s location via GPS data, and then checking the local weather station to see if there was recent precipitation—in these instances, rainfall had occurred within three days and it is likely the bighorn was drinking from water in a rock catchment.

**Appendix S2: Testing multiple metrics of body temperature variation.**

Increased body temperature variation, referred to as heterothermy, is associated with energetic trade-offs and may be associated with fewer pregnancies (Maloney *et al.*, 2017), dehydration (Fuller *et al.*, 2021), and energetically compromised individuals (Hetem *et al.*, 2016; Botha *et al.*, 2023). The heterothermy index (HI) is a standardized metric for the amount of body temperature variation experienced over a given timeframe and allows comparisons across species (Boyles *et al.*, 2011). It is calculated by first calculating the modal body temperature for each individual as a proxy for optimal body temperature, then calculating body temperature standard deviation relative to the mode instead of the mean over the period of interest (Boyles *et al.*, 2011). For the daily heterothermy index used in all analyses, the modal body temperature was calculated separately for each individual on each calendar day. Amplitude is calculated by subtracting the daily minimum from the maximum, whereas IQR measures the spread of the middle 50% of the daily data and is robust against outliers. We report amplitude values in the manuscript.

While each of the body temperature variation metrics is calculated slightly differently, each performed similarly. For the linear mixed-effects model, each body temperature variation metric produced the same pattern of results (Table S3b). Body temperature variation was elevated from February through July and animals in MARB showed higher body temperature variation than any other range across all three metrics (LME amplitude: β = 0.491, t = 3.675, p = 0.001; IQR: β = 0.173, t = 2.878, p = 0.008; HI: β = 0.148, t = 3.220, p = 0.003). Furthermore, Sex x YearMonth interactions were negative from February through June for amplitude and through July for IQR, and HI (LME amplitude: β = −0.324 to −0.117, t = −7.40 to −2.63, all p ≤ 0.009; LME IQR: β = −0.186 to −0.064, t = −9.30 to −3.18, all p ≤ 0.002; LME HI: β = −0.130 to −0.042, t = −8.55 to −2.73, all p ≤ 0.006), indicating that females exhibited greater body temperature variation than males across all three metrics during this period. All three body variation metrics were strongly associated with an increase in mortality risk (Figure S1, Table S4b). Per 0.1-unit increase in HI, the hazard of mortality (HR) increased approximately 4.40-fold (HR = exp (14.807 * 0.1) = 4.40; LRT χ²(1) = 14.27, p-value < 0.001). Per 0.1 °C increase in IQR the hazard of mortality increased approximately 3.64 fold (HR = exp (12.908 * 0.1) = 3.64; LRT χ²(1) = 13.36, p-value < 0.001). Per 0.1 °C increase in amplitude, the hazard of mortality increased by ~1.68-fold (HR = exp(5.215 * 0.1) = 1.68; LRT χ²(1) = 15.81, p < 0.001).

Figure S1: Partial effects plots exhibiting the predicted probabilities of mortality of desert bighorn sheep (mortality event = 1) for each body temperature variation metric from Nov. 2020-Dec. 2021. Plot A shows the significant relationship between increased amplitude (°C) and increased risk of mortality. Plot B shows the significant relationship between increased Heterothermy Index and increased risk of mortality. Plot C shows the significant relationship between increased interquartile range (IQR, °C) and increased risk of mortality. Predictions were generated using the observed values for each focal covariate while all other fixed-effect variables were held constant. Shading represents 95% confidence intervals.

**Literature Cited Appendices 1 and 2**

Beatty DT, Barnes A, Taylor E, Maloney SK (2008) Do changes in feed intake or ambient temperature cause changes in cattle rumen temperature relative to core temperature? *J Therm Biol* 33: 12–19.

Botha A, Fuller A, Beechler BR, Combrink HJ, Jolles AE, Maloney SK, Hetem RS (2023) Cold and Hungry: Heterothermy Is Associated with Low Leptin Levels in a Bulk Grazer during a Drought. *Physiol Biochem Zool* 96: 342–355.

Boyles JG, Smit B, McKechnie AE (2011) A new comparative metric for estimating heterothermy in endotherms. *Physiol Biochem Zool* 84: 115–123.

Fuller A, Mitchell D, Maloney SK, Hetem RS, Fonsêca VFC, Meyer LCR, Van De Ven TMFN, Snelling EP (2021) How dryland mammals will respond to climate change: the effects of body size, heat load and a lack of food and water. *J Exp Biol* 224: jeb238113.

Herberg AM, St-Louis V, Carstensen M, Fieberg J, Thompson DP, Crouse JA, Forester JD (2018a) Calibration of a rumen bolus to measure continuous internal body temperature in moose. *Wildl Soc Bull* 42: 328–337.

Herberg AM, St-Louis V, Carstensen M, Fieberg J, Thompson DP, Crouse JA, Forester JD (2018b) Calibration of a rumen bolus to measure continuous internal body temperature in moose. *Wildl Soc Bull* 42: 328–337.

Hetem RS, Maloney SK, Fuller A, Mitchell D (2016) Heterothermy in large mammals: inevitable or implemented? *Biol Rev* 91: 187–205.

Maloney SK, Marsh MK, McLeod SR, Fuller A (2017) Heterothermy is associated with reduced fitness in wild rabbits. *Biol Lett* 13: 20170521.

Thompson DP, Crouse JA, Barboza PS, Spathelf MO, Herberg AM, Parker SD, Morris MA (2021) Behaviour influences thermoregulation of boreal moose during the warm season. *Conserv Physiol* 9: 1–12.

Vesterdorf K, Beatty DT, Barnes A, Maloney SK (2022) Rumen temperature is a reliable proxy of core body temperature in sheep (Ovis aries). *Anim Prod Sci* 62: 1671–1682.

Table S1: The minimum and maximum elevation and the difference (amplitude) for each of the six mountain ranges (populations).

| Range | Minimum Elevation (m) | Maximum Elevation (m) | Amplitude Elevation (m) |
| --- | --- | --- | --- |
| SODA | 276 | 738 | 462 |
| MARB | 241 | 1163 | 922 |
| ODKM | 414 | 1498 | 1084 |
| CMPR | 757 | 1769 | 1012 |
| WDHK | 946 | 1885 | 939 |
| NROM | 552 | 1918 | 1366 |

Figure S2: The survival status and available reticulum implant transmitter (RIT) data for the 43 individual desert bighorn sheep (BHS_ID) during the study period of November 2020 through December 2021. During this period eight female bighorn died. These eight mortalities occurred throughout the study period; cause of least four of the mortalities was suspected predation, one was suspected malnourishment and/or disease, and two were unknown. Three RITS failed between seven and ten months but were still included in the analyses. Additionally, one ram was recaptured in November 2021 due to a collar communication issue, enabling the recovery of the GPS collar and thereby the RIT data.

Table S2: Expected drinking days per calendar month (December 2020-November 2021) for collared desert bighorn in six mountain ranges within the Mojave Desert of California. Entries are marginal means with 95% confidence intervals, expressed as the expected number of days in a calendar month on which a randomly selected individual drank at least once per day.

| Year Month | WDHK | CMPR | NROM | ODKM | Marb | Soda |
| --- | --- | --- | --- | --- | --- | --- |
| 2020-12 | 1.0 (0.4-2.1) | 3.5 (2.1-5.6) | 10.6 (8.2-13.2) | 7.8 (5.5-10.5) | 11.2 (8.4-14.2) | 10.0 (7.5-13.0) |
| 2021-01 | 5.9 (4.0-8.3) | 7.5 (5.0-10.8) | 11.7 (9.3-14.4) | 10.1 (7.5-13.0) | 10.1 (7.6-13.1) | 9.3 (6.9-12.2) |
| 2021-02 | 8.3 (6.1-11.0) | 12.0 (8.8-15.4) | 8.9 (6.8-11.3) | 5.6 (3.8-8.0) | 7.4 (5.3-10.0) | 5.8 (3.9-8.3) |
| 2021-03 | 4.6 (3.0-6.8) | 12.7 (9.3-16.4) | 5.6 (4.0-7.7) | 3.5 (2.2-5.4) | 6.1 (4.2-8.6) | 7.8 (5.4-10.8) |
| 2021-04 | 1.6 (0.9-3.0) | 7.8 (5.2-11.0) | 5.9 (4.3-8.0) | 3.5 (2.2-5.4) | 7.7 (5.5-10.4) | 9.0 (6.4-12.2) |
| 2021-05 | 3.3 (2.1-5.2) | 9.9 (6.9-13.4) | 7.8 (5.8-10.2) | 8.6 (6.3-11.4) | 11.1 (8.3-14.3) | 8.4 (5.9-11.6) |
| 2021-06 | 6.7 (4.7-9.3) | 14.1 (10.6-17.7) | 13.7 (11.1-16.4) | 10.8 (8.2-13.7) | 15.3 (12.2-18.4) | 9.8 (7.1-13.1) |
| 2021-07 | 14.2 (11.1-17.4) | 20.6 (17.1-23.7) | 14.9 (12.2-17.6) | 13.0 (10.1-16.0) | 18.9 (15.6-21.9) | 13.1 (9.8-16.5) |
| 2021-08 | 7.4 (5.2-10.3) | 15.4 (11.8-19.0) | 9.3 (7.1-11.9) | 10.3 (7.6-13.3) | 15.9 (12.5-19.2) | 10.0 (7.2-13.3) |
| 2021-09 | 9.7 (7.1-12.7) | 13.9 (10.4-17.4) | 10.2 (7.9-12.8) | 5.1 (3.4-7.6) | 15.0 (11.8-18.3) | 9.8 (7.1-13.1) |
| 2021-10 | 9.8 (7.1-13.0) | 13.4 (9.9-17.0) | 10.1 (7.8-12.7) | 7.5 (5.2-10.3) | 12.5 (9.3-16.0) | 7.9 (5.3-11.1) |
| 2021-11 | 8.1 (5.6-11.2) | 8.0 (5.4-11.2) | 6.4 (4.6-8.6) | 5.6 (3.8-8.2) | 8.0 (5.5-11.3) | 5.7 (3.6-8.6) |

Table S3a: Results of four linear mixed-effects models (LME) for daily body temperature metrics in desert bighorn sheep (*Ovis canadensis nelsoni*; n = 43 individuals, 14,006 animal-days): Median (°C), Maximum (°C), Minimum (°C), Amplitude (°C; daily max – min). Fixed effects: Sex(male = ‘SexM’; female = reference), Mountain Range (reference = ODKM), Age class (reference = prime-age adult, 2-5 yr), Month (reference = December 2020), and BMIsc (centered linear and quadratic). All models include a random intercept for individual (BHS_ID) and a continuous-time AR(1) residual correlation structure (corCAR1), fit by REML. Coefficients (β) are conditional effects relative to the reference animal (female, prime-age adult, ODKM, December 2020, mean BMIsc). T-values can be calculated as β/SE. R²m = marginal R² (fixed effects only); R²c = conditional R² (fixed + random; Nakagawa & Schielzeth 2013). σ²ID = random intercept variance; σ²res = residual variance; ρ = CAR(1) autocorrelation parameter.

| **Term** | **Median** | | | **Maximum** | | | **Minimum** | | | **Amplitude** | | |
| --- | --- | --- | --- | --- | --- | --- | --- | --- | --- | --- | --- | --- |
|  | ***β*** | ***SE*** | ***p*** | ***β*** | ***SE*** | ***p*** | ***β*** | ***SE*** | ***p*** | ***β*** | ***SE*** | ***p*** |
| ***Parametric fixed effects*** | | | | | | | | | | | | |
| Intercept | 38.787 | 0.054 | < 0.001 | 39.444 | 0.073 | < 0.001 | 38.140 | 0.087 | < 0.001 | 1.303 | 0.102 | < 0.001 |
| Sex (Male) | -0.051 | 0.088 | 0.565 | -0.087 | 0.117 | 0.467 | -0.087 | 0.141 | 0.541 | 0.002 | 0.165 | 0.990 |
| Range: CMPR | -0.046 | 0.078 | 0.561 | 0.040 | 0.105 | 0.705 | -0.141 | 0.126 | 0.273 | 0.183 | 0.148 | 0.226 |
| Range: MARB | 0.015 | 0.070 | 0.837 | 0.211 | 0.094 | 0.033 | -0.280 | 0.114 | 0.021 | 0.491 | 0.134 | 0.001 |
| Range: NROM | 0.127 | 0.066 | 0.065 | 0.167 | 0.089 | 0.070 | 0.068 | 0.107 | 0.530 | 0.098 | 0.126 | 0.442 |
| Range: SODA | 0.112 | 0.074 | 0.141 | 0.268 | 0.099 | 0.012 | -0.099 | 0.119 | 0.412 | 0.367 | 0.140 | 0.014 |
| Range: WDHK | 0.100 | 0.067 | 0.148 | 0.159 | 0.090 | 0.088 | -0.009 | 0.109 | 0.937 | 0.168 | 0.128 | 0.200 |
| Age class 1 (≤2 yr) | 0.135 | 0.061 | 0.034 | 0.185 | 0.081 | 0.031 | 0.216 | 0.098 | 0.037 | -0.031 | 0.115 | 0.789 |
| Age class 3 (≥6 yr) | 0.045 | 0.044 | 0.320 | 0.041 | 0.059 | 0.489 | 0.067 | 0.071 | 0.352 | -0.027 | 0.084 | 0.748 |
| ***Month (reference = December 2020)*** | | | | | | | | | | | | |
| January | -0.010 | 0.014 | 0.465 | 0.034 | 0.020 | 0.097 | -0.050 | 0.019 | 0.008 | 0.082 | 0.022 | < 0.001 |
| February | -0.005 | 0.015 | 0.716 | 0.126 | 0.021 | < 0.001 | -0.233 | 0.020 | < 0.001 | 0.364 | 0.023 | < 0.001 |
| March | 0.001 | 0.014 | 0.947 | 0.182 | 0.021 | < 0.001 | -0.367 | 0.019 | < 0.001 | 0.553 | 0.023 | < 0.001 |
| April | 0.072 | 0.015 | < 0.001 | 0.196 | 0.021 | < 0.001 | -0.270 | 0.020 | < 0.001 | 0.467 | 0.023 | < 0.001 |
| May | 0.078 | 0.015 | < 0.001 | 0.241 | 0.021 | < 0.001 | -0.260 | 0.020 | < 0.001 | 0.506 | 0.023 | < 0.001 |
| June | 0.097 | 0.015 | < 0.001 | 0.317 | 0.021 | < 0.001 | -0.161 | 0.020 | < 0.001 | 0.488 | 0.023 | < 0.001 |
| July | 0.135 | 0.015 | < 0.001 | 0.343 | 0.021 | < 0.001 | -0.063 | 0.020 | 0.001 | 0.403 | 0.023 | < 0.001 |
| August | 0.074 | 0.015 | < 0.001 | 0.286 | 0.022 | < 0.001 | -0.123 | 0.020 | < 0.001 | 0.412 | 0.024 | < 0.001 |
| September | 0.024 | 0.015 | 0.118 | 0.136 | 0.022 | < 0.001 | -0.084 | 0.020 | < 0.001 | 0.223 | 0.024 | < 0.001 |
| October | 0.005 | 0.015 | 0.764 | 0.069 | 0.022 | 0.002 | -0.080 | 0.021 | < 0.001 | 0.148 | 0.024 | < 0.001 |
| November | -0.008 | 0.016 | 0.603 | 0.015 | 0.023 | 0.501 | -0.014 | 0.021 | 0.521 | 0.032 | 0.025 | 0.208 |
| ***Body size (BMIsc)*** | | | | | | | | | | | | |
| BMIsc (linear) | 0.002 | 0.003 | 0.427 | -0.002 | 0.004 | 0.641 | 0.006 | 0.005 | 0.188 | -0.008 | 0.005 | 0.146 |
| BMIsc (quadratic) | -0.001 | 0.000 | 0.082 | 0.000 | 0.000 | 0.337 | -0.001 | 0.000 | 0.144 | 0.000 | 0.001 | 0.555 |
| ***Sex × Range interactions*** | | | | | | | | | | | | |
| Sex × CMPR | 0.110 | 0.125 | 0.389 | 0.057 | 0.168 | 0.735 | 0.257 | 0.203 | 0.216 | -0.202 | 0.238 | 0.405 |
| Sex × MARB | 0.101 | 0.122 | 0.418 | 0.090 | 0.163 | 0.585 | 0.100 | 0.198 | 0.618 | -0.010 | 0.232 | 0.965 |
| Sex × NROM | -0.163 | 0.117 | 0.175 | -0.182 | 0.156 | 0.255 | -0.128 | 0.189 | 0.506 | -0.054 | 0.222 | 0.811 |
| Sex × SODA | -0.150 | 0.154 | 0.340 | -0.080 | 0.206 | 0.701 | -0.128 | 0.249 | 0.611 | 0.050 | 0.292 | 0.865 |
| Sex × WDHK | 0.236 | 0.188 | 0.221 | 0.141 | 0.253 | 0.582 | 0.382 | 0.304 | 0.219 | -0.245 | 0.357 | 0.498 |
| ***Sex × Month interactions*** | | | | | | | | | | | | |
| Sex × January | 0.074 | 0.027 | 0.006 | 0.079 | 0.040 | 0.045 | 0.050 | 0.036 | 0.169 | 0.025 | 0.043 | 0.568 |
| Sex × February | 0.017 | 0.028 | 0.541 | -0.047 | 0.041 | 0.251 | 0.142 | 0.038 | < 0.001 | -0.189 | 0.045 | < 0.001 |
| Sex × March | 0.062 | 0.028 | 0.026 | -0.053 | 0.040 | 0.183 | 0.270 | 0.037 | < 0.001 | -0.324 | 0.044 | < 0.001 |
| Sex × April | 0.026 | 0.028 | 0.359 | 0.019 | 0.040 | 0.634 | 0.227 | 0.037 | < 0.001 | -0.212 | 0.044 | < 0.001 |
| Sex × May | 0.017 | 0.028 | 0.546 | -0.079 | 0.040 | 0.050 | 0.117 | 0.037 | 0.002 | -0.201 | 0.044 | < 0.001 |
| Sex × June | -0.036 | 0.028 | 0.199 | -0.061 | 0.041 | 0.132 | 0.053 | 0.038 | 0.158 | -0.117 | 0.044 | 0.009 |
| Sex × July | -0.137 | 0.028 | < 0.001 | -0.157 | 0.040 | < 0.001 | -0.109 | 0.037 | 0.004 | -0.048 | 0.044 | 0.278 |
| Sex × August | -0.148 | 0.028 | < 0.001 | -0.056 | 0.041 | 0.166 | -0.109 | 0.038 | 0.004 | 0.049 | 0.044 | 0.266 |
| Sex × September | -0.082 | 0.028 | 0.004 | -0.067 | 0.041 | 0.104 | -0.136 | 0.038 | < 0.001 | 0.068 | 0.045 | 0.130 |
| Sex × October | -0.055 | 0.028 | 0.052 | -0.063 | 0.041 | 0.126 | -0.072 | 0.038 | 0.059 | 0.011 | 0.045 | 0.806 |
| Sex × November | -0.053 | 0.029 | 0.069 | -0.010 | 0.042 | 0.817 | -0.050 | 0.039 | 0.199 | 0.035 | 0.046 | 0.445 |
| **Model fit statistics** | | | | | | | | | | | | |
| **R²m (marginal R²)** | 0.168 | | | 0.143 | | | 0.215 | | | 0.242 | | |
| **R²c (conditional R²)** | 0.359 | | | 0.251 | | | 0.400 | | | 0.379 | | |
| **σ²ID (random intercept var.)** | 0.010 | | | 0.018 | | | 0.026 | | | 0.036 | | |
| **σ²res (residual var.)** | 0.033 | | | 0.124 | | | 0.086 | | | 0.166 | | |
| **ρ – CAR(1) phi** | 0.526 | | | 0.246 | | | 0.354 | | | 0.189 | | |

Table S3b. Results of two additional body temperature variation models: Interquartile Range (IQR; °C) and Heterothermy Index (HI). Model structure identical to Table S3a, see notes for interpretation.

| **Term** | **IQR** | | | **HI** | | |
| --- | --- | --- | --- | --- | --- | --- |
|  | ***β*** | ***SE*** | ***p*** | ***β*** | ***SE*** | ***p*** |
| ***Parametric fixed effects*** | | | | | | |
| Intercept | 0.395 | 0.046 | < 0.001 | 0.318 | 0.035 | < 0.001 |
| Sex (Male) | 0.054 | 0.074 | 0.473 | 0.028 | 0.057 | 0.627 |
| Range: CMPR | 0.078 | 0.067 | 0.254 | 0.063 | 0.051 | 0.230 |
| Range: MARB | 0.173 | 0.060 | 0.008 | 0.148 | 0.046 | 0.003 |
| Range: NROM | 0.017 | 0.057 | 0.770 | 0.021 | 0.043 | 0.629 |
| Range: SODA | 0.085 | 0.063 | 0.187 | 0.080 | 0.048 | 0.111 |
| Range: WDHK | 0.073 | 0.058 | 0.215 | 0.061 | 0.044 | 0.177 |
| Age class 1 (≤2 yr) | -0.039 | 0.052 | 0.463 | -0.032 | 0.040 | 0.433 |
| Age class 3 (≥6 yr) | -0.014 | 0.038 | 0.712 | -0.017 | 0.029 | 0.569 |
| ***Month (reference = December 2020)*** | | | | | | |
| January | 0.039 | 0.010 | < 0.001 | 0.028 | 0.008 | < 0.001 |
| February | 0.184 | 0.011 | < 0.001 | 0.143 | 0.008 | < 0.001 |
| March | 0.308 | 0.010 | < 0.001 | 0.226 | 0.008 | < 0.001 |
| April | 0.261 | 0.011 | < 0.001 | 0.208 | 0.008 | < 0.001 |
| May | 0.281 | 0.011 | < 0.001 | 0.216 | 0.008 | < 0.001 |
| June | 0.288 | 0.011 | < 0.001 | 0.222 | 0.008 | < 0.001 |
| July | 0.227 | 0.011 | < 0.001 | 0.179 | 0.008 | < 0.001 |
| August | 0.226 | 0.011 | < 0.001 | 0.178 | 0.008 | < 0.001 |
| September | 0.133 | 0.011 | < 0.001 | 0.107 | 0.008 | < 0.001 |
| October | 0.079 | 0.011 | < 0.001 | 0.070 | 0.009 | < 0.001 |
| November | 0.009 | 0.011 | 0.422 | 0.021 | 0.009 | 0.015 |
| ***Body size (BMIsc)*** | | | | | | |
| BMIsc (linear) | -0.004 | 0.002 | 0.149 | -0.003 | 0.002 | 0.109 |
| BMIsc (quadratic) | 0.000 | 0.000 | 0.419 | 0.000 | 0.000 | 0.300 |
| ***Sex × Range interactions*** | | | | | | |
| Sex × CMPR | -0.096 | 0.107 | 0.378 | -0.075 | 0.082 | 0.367 |
| Sex × MARB | -0.058 | 0.105 | 0.583 | -0.032 | 0.080 | 0.689 |
| Sex × NROM | -0.013 | 0.100 | 0.896 | -0.008 | 0.077 | 0.918 |
| Sex × SODA | 0.045 | 0.132 | 0.734 | 0.045 | 0.101 | 0.661 |
| Sex × WDHK | -0.141 | 0.161 | 0.390 | -0.129 | 0.123 | 0.303 |
| ***Sex × Month interactions*** | | | | | | |
| Sex × January | 0.010 | 0.020 | 0.627 | 0.023 | 0.015 | 0.127 |
| Sex × February | -0.085 | 0.020 | < 0.001 | -0.069 | 0.016 | < 0.001 |
| Sex × March | -0.186 | 0.020 | < 0.001 | -0.130 | 0.015 | < 0.001 |
| Sex × April | -0.120 | 0.020 | < 0.001 | -0.092 | 0.015 | < 0.001 |
| Sex × May | -0.106 | 0.020 | < 0.001 | -0.069 | 0.015 | < 0.001 |
| Sex × June | -0.089 | 0.020 | < 0.001 | -0.059 | 0.015 | < 0.001 |
| Sex × July | -0.064 | 0.020 | 0.002 | -0.042 | 0.015 | 0.006 |
| Sex × August | -0.031 | 0.020 | 0.127 | -0.010 | 0.015 | 0.518 |
| Sex × September | 0.028 | 0.020 | 0.168 | 0.015 | 0.016 | 0.348 |
| Sex × October | 0.007 | 0.020 | 0.724 | 0.001 | 0.016 | 0.942 |
| Sex × November | 0.016 | 0.021 | 0.461 | 0.007 | 0.016 | 0.671 |
| **Model fit statistics** | | | | | | |
| **R²m (marginal R²)** | 0.217 | | | 0.233 | | |
| **R²c (conditional R²)** | 0.338 | | | 0.355 | | |
| **σ²ID (random intercept var.)** | 0.007 | | | 0.004 | | |
| **σ²res (residual var.)** | 0.041 | | | 0.023 | | |
| **ρ – CAR(1) phi** | 0.104 | | | 0.117 | | |

Figure S3: Pearson’s correlation matrix among the candidate fixed effects used in the survival analysis: Age, daily body temperature amplitude (b.amplitude), maximum (b.max_temp), median (b.median_temp), and minimum (b.min_temp). The heterothermy index (HI.Daily) and interquartile range (b.IQR) are also included to show the high correlation with amplitude. Tile labels give Pearson’s *r-value* and color indicates direction and magnitude. Body temperature metrics are strongly collinear (e.g., amplitude verse minimum: *r* = -0.77; maximum vs. median: *r* = 0.73), supporting our choice to fit separate single-metric models to avoid multicollinearity.

Table S4a: Mixed-effects Cox proportional hazard model results for daily body temperature metrics in desert bighorn sheep (n = 43 individuals, 165 100-day intervals, 8 mortality events). Each column represents a separate *coxme* model with fixed effects of centered age (mean = 4.8 years), one body temperature metric (Amplitude, Median, Maximum, and Minimum), and random intercepts for individual (BHS_ID) and mountain range (Range). Coefficients are log-hazard estimates with standard errors (SE). Significance of each body temperature term was assessed via likelihood ratio tests (LRT) comparing the metric model to the age-only baseline model. Superscripts: *** p <0.001; σ = random effect standard deviation. Proportional hazard assumption was verified via Schoenfeld residuals (*cox.zph*; all p > 0.05).

| **Term** | **Age only** | | **Amplitude** | | **Median** | | **Maximum** | | **Minimum** | |
| --- | --- | --- | --- | --- | --- | --- | --- | --- | --- | --- |
|  | ***β*** | ***SE*** | ***β*** | ***SE*** | ***β*** | ***SE*** | ***β*** | ***SE*** | ***β*** | ***SE*** |
| Age (centered) | 0.262 | (0.157) | 0.105 | (0.136) | 0.226 | (0.151) | 0.250 | (0.153) | 0.105 | (0.135) |
| Amplitude | — |  | 5.215*** | (1.481) | — |  | — |  | — |  |
| Median Tb | — |  | — |  | -2.956 | (2.883) | — |  | — |  |
| Maximum Tb | — |  | — |  | — |  | 2.710 | (2.233) | — |  |
| Minimum Tb | — |  | — |  | — |  | — |  | -5.926*** | (1.700) |
| **Model fit statistics** | | | | | | | | | | |
| **LRT χ²** | — |  | 15.811 |  | 1.087 |  | 1.447 |  | 14.833 |  |
| **LRT p-value** | 0.086 |  | < 0.001 |  | 0.297 |  | 0.229 |  | < 0.001 |  |
| **σ (BHS_ID)** | 0.574 |  | 0.336 |  | 0.701 |  | 0.020 |  | 0.020 |  |
| **σ (Range)** | 0.710 |  | 0.103 |  | 0.553 |  | 0.505 |  | 0.020 |  |
| **n (intervals)** | 165 |  | 165 |  | 165 |  | 165 |  | 165 |  |
| **n (events)** | 8 |  | 8 |  | 8 |  | 8 |  | 8 |  |
| **n (individuals)** | 43 |  | 43 |  | 43 |  | 43 |  | 43 |  |

Table S4b: Mixed-effects Cox proportional hazard model results for body temperature variation metrics. Model structure identical to Table S5a. HI = Heterothermy Index; IQR = Interquartile Range. All three variation metrics—Amplitude, HI, and IQR –were significantly associated with increased mortality risk, confirming the robustness of the heterothermy-mortality association across alternative metrics.

| **Term** | **Age only** | | **Amplitude** | | **HI** | | **IQR** | |
| --- | --- | --- | --- | --- | --- | --- | --- | --- |
|  | ***β*** | ***SE*** | ***β*** | ***SE*** | ***β*** | ***SE*** | ***β*** | ***SE*** |
| Age (centered) | 0.262 | (0.157) | 0.105 | (0.136) | 0.114 | (0.133) | 0.083 | (0.136) |
| Amplitude | — |  | 5.215*** | (1.481) | — |  | — |  |
| Heterothermy Index | — |  | — |  | 14.807*** | (4.143) | — |  |
| IQR | — |  | — |  | — |  | 12.908*** | (3.910) |
| **Model fit statistics** | | | | | | | | |
| **LRT χ²** | — |  | 15.811 |  | 14.272 |  | 13.359 |  |
| **LRT p-value** | 0.086 |  | < 0.001 |  | < 0.001 |  | < 0.001 |  |
| **σ (BHS_ID)** | 0.574 |  | 0.336 |  | 0.020 |  | 0.020 |  |
| **σ (Range)** | 0.710 |  | 0.103 |  | 0.400 |  | 0.466 |  |
| **n (intervals)** | 165 |  | 165 |  | 165 |  | 165 |  |
| **n (events)** | 8 |  | 8 |  | 8 |  | 8 |  |
| **n (individuals)** | 43 |  | 43 |  | 43 |  | 43 |  |
